# Supplementary material for: A fault-intrusion-tolerant system and deadline-aware algorithm for scheduling scientific workflow in the cloud
Source: PeerJ Comput Sci. 2021 Nov 2;7:e747. doi: 10.7717/peerj-cs.747 (PMC8576554; doi:10.7717/peerj-cs.747)
Supplement: Supplemental Information 6 [file peerj-cs-07-747-s006.zip › Myproject1.1/docs/org/cloudbus/cloudsim/Log.html]

Log


JavaScript is disabled on your browser.


- Overview
- Package
- Class
- Use
- Tree
- Deprecated
- Index
- Help

- Prev Class
- Next Class

- Frames
- No Frames

- All Classes

- Summary:
- Nested |
- Field |
- Constr |
- Method

- Detail:
- Field |
- Constr |
- Method


org.cloudbus.cloudsim

## Class Log

- java.lang.Object
- - org.cloudbus.cloudsim.Log

- ---

    

  ```
  public class Log
  extends java.lang.Object
  ```

  The Log class used for performing loggin of the simulation process. It provides the ability to
  substitute the output stream by any OutputStream subclass.

  Since:
  :   CloudSim Toolkit 2.0

- - ### Constructor Summary

    Constructors

    Constructor and Description

    `Log()`
  - ### Method Summary

    Methods

    | Modifier and Type | Method and Description |
    | --- | --- |
    | `static void` | `disable()` Disables the output. |
    | `static void` | `enable()` Enables the output. |
    | `static void` | `format(java.lang.String format, java.lang.Object... args)` Prints a string formated as in String.format(). |
    | `static void` | `formatLine(java.lang.String format, java.lang.Object... args)` Prints a line formated as in String.format(). |
    | `static java.io.OutputStream` | `getOutput()` Gets the output. |
    | `static boolean` | `isDisabled()` Checks if the output is disabled. |
    | `static void` | `print(java.lang.Object message)` Prints the message passed as a non-String object. |
    | `static void` | `print(java.lang.String message)` Prints the message. |
    | `static void` | `printLine()` Prints the empty line. |
    | `static void` | `printLine(java.lang.Object message)` Prints the line passed as a non-String object. |
    | `static void` | `printLine(java.lang.String message)` Prints the line. |
    | `static void` | `setDisabled(boolean _disabled)` Sets the disable output flag. |
    | `static void` | `setOutput(java.io.OutputStream _output)` Sets the output. |

    - ### Methods inherited from class java.lang.Object

      `clone, equals, finalize, getClass, hashCode, notify, notifyAll, toString, wait, wait, wait`

- - ### Constructor Detail


    - #### Log

      ```
      public Log()
      ```
  - ### Method Detail


    - #### print

      ```
      public static void print(java.lang.String message)
      ```

      Prints the message.

      Parameters:
      :   `message` - the message


    - #### print

      ```
      public static void print(java.lang.Object message)
      ```

      Prints the message passed as a non-String object.

      Parameters:
      :   `message` - the message


    - #### printLine

      ```
      public static void printLine(java.lang.String message)
      ```

      Prints the line.

      Parameters:
      :   `message` - the message


    - #### printLine

      ```
      public static void printLine()
      ```

      Prints the empty line.


    - #### printLine

      ```
      public static void printLine(java.lang.Object message)
      ```

      Prints the line passed as a non-String object.

      Parameters:
      :   `message` - the message


    - #### format

      ```
      public static void format(java.lang.String format,
                java.lang.Object... args)
      ```

      Prints a string formated as in String.format().

      Parameters:
      :   `format` - the format
      :   `args` - the args


    - #### formatLine

      ```
      public static void formatLine(java.lang.String format,
                    java.lang.Object... args)
      ```

      Prints a line formated as in String.format().

      Parameters:
      :   `format` - the format
      :   `args` - the args


    - #### setOutput

      ```
      public static void setOutput(java.io.OutputStream _output)
      ```

      Sets the output.

      Parameters:
      :   `_output` - the new output


    - #### getOutput

      ```
      public static java.io.OutputStream getOutput()
      ```

      Gets the output.

      Returns:
      :   the output


    - #### setDisabled

      ```
      public static void setDisabled(boolean _disabled)
      ```

      Sets the disable output flag.

      Parameters:
      :   `_disabled` - the new disabled


    - #### isDisabled

      ```
      public static boolean isDisabled()
      ```

      Checks if the output is disabled.

      Returns:
      :   true, if is disable


    - #### disable

      ```
      public static void disable()
      ```

      Disables the output.


    - #### enable

      ```
      public static void enable()
      ```

      Enables the output.


- Overview
- Package
- Class
- Use
- Tree
- Deprecated
- Index
- Help

- Prev Class
- Next Class

- Frames
- No Frames

- All Classes

- Summary:
- Nested |
- Field |
- Constr |
- Method

- Detail:
- Field |
- Constr |
- Method
